# The impact of intermittent energy restriction on women’s health

**DOI:** 10.1017/S0029665125000059

**Published:** 2025-02-11

**Authors:** Michelle Harvie, Mai Haiba

**Affiliations:** 1 Division of Cancer Sciences, The University of Manchester, Manchester, UK; 2 Department of Dietetics, Wythenshawe Hospital, Manchester University NHS Foundation Trust, Manchester, M23 9LT, UK; 3 Manchester Breast Centre, Oglesby Cancer Research Centre, The Christie NHS Foundation Trust, University of Manchester, Manchester, UK

**Keywords:** Intermittent energy restriction, time restricted eating, women’s health, weight loss

## Abstract

Intermittent energy restricted diets are used amongst women with overweight and obesity and a healthy weight. For those with overweight and obesity weight control is typically achieved through daily energy restriction (DER) which has reduced adherence and attenuated metabolic benefits over time. Several intermittent energy restriction (IER) regimens have been developed aiming to promote maintained weight loss and additional weight independent metabolic benefits including the 5:2 diet, alternate day fasting (ADF) and time restricted eating (TRE). This review summarises the potential benefits or harms of these regimens for managing women’s health. 5:2 and ADF diets have equivalent long term (≥6-month) adherence, weight loss and metabolic benefits to DER. Current limited evidence suggests IER is a safe weight loss intervention for women which does not affect reproductive or bone health, increase eating disorders or disturb sleep. Adherence and weight loss with both IER and DER are lower amongst younger women compared to older women and men. Weight loss with ADF and TRE have respectively improved symptoms of polycystic ovarian syndrome and premenstrual syndrome but there is no evidence of weight independent effects of IER on these conditions. There is little evidence of the benefits and/or harms of IER amongst healthy weight women in whom there is a greater potential for adverse effects on reproductive and bone health, fat free mass, eating disorders and sleep. Further research benefits of IER for weight control and metabolic health as well as harms is required.

## Incidence and consequences of overweight and obesity amongst women

Overweight and obesity pose a major health problem for women. In 2023 an estimated 61% of adult females in the UK were living with either overweight or obesity. Twenty-nine percent of these were living with obesity, which had increased from 26% in 2013 and 23% in 2003 (1). Increasing rates of obesity are evident among younger females (11-15 years) where currently 20% living with obesity compared to 17% in the year 2013 and 6.6% in year 2003(2).

There is an ethnic disparity with higher rates of obesity amongst women from black Caribbean (44%), Pakistani (33%) and black African (37%) backgrounds compared to women in the white British population (28%) in 2019(3). These obesity estimates are based on BMI > 30kg / m^2^ so are likely to underestimate the true impact of obesity related health conditions within ethnic groups since these groups experience obesity related comorbidities at a lower BMI than white women i.e. overweight is defined at 23-27 kg/m^2^ and obesity at 27 kg/m^2^ in these groups(4). Women from socially deprived backgrounds experience higher rates of obesity. In 2021 40% of women in the most deprived areas were living with obesity as compared to 19% of women in the least deprived areas(5).

Overweight and obesity amongst women are linked to higher risk of 11 cancers including breast and endometrial cancer(6). Obesity poses a greater cancer burden for women amongst whom 55% of cancers are related to obesity compared to only 24% of cancers among men(7). Obesity also contributes to other health issues such as cardiovascular disease, type 2 diabetes, osteoarthritis and reproductive complications including infertility which significantly impacts their quality of life as well as increasing healthcare costs(8, 9).

## Intermittent energy restricted diets

**Figure 1:**
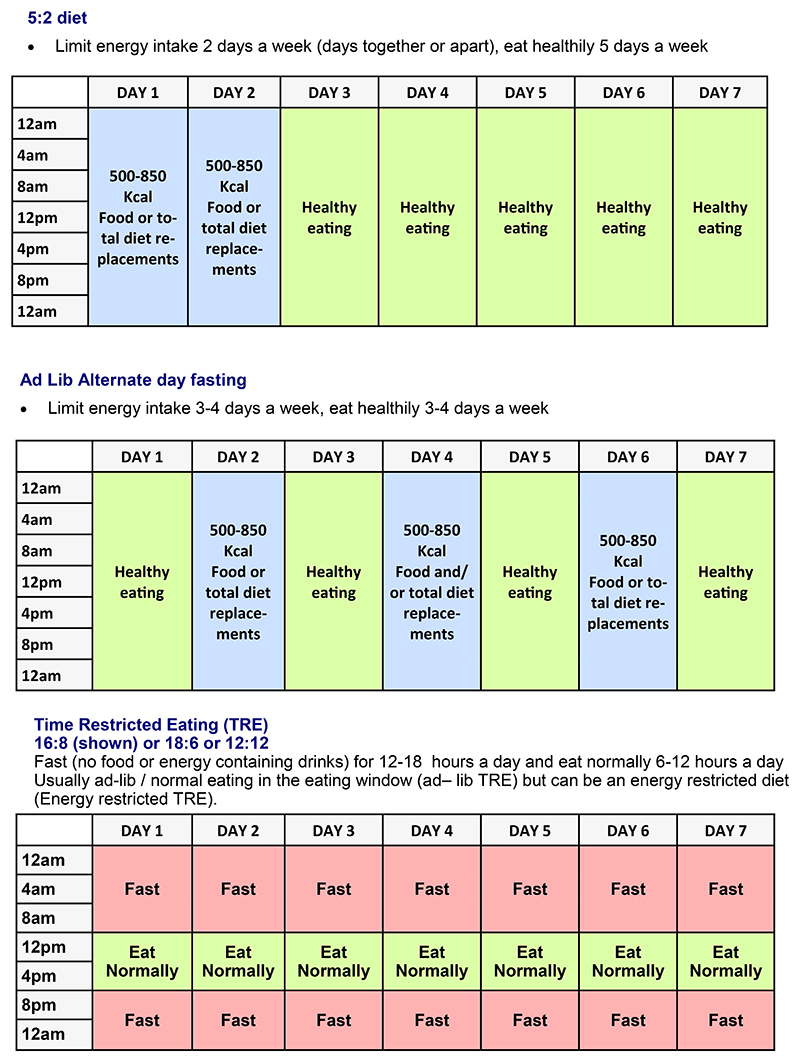
Popular regimes for intermittent energy restriction regimens

Energy restricted diets are the cornerstone of the management of overweight and obesity and weight related metabolic disease. Current evidence based guidelines recommend daily energy restriction, typically a 600 kcal energy deficit for sustainable weight loss(4). Daily energy restriction (DER) can be effective for weight loss but has reduced adherence over time. Also, the metabolic benefits of DER and weight loss are attenuated once weight is reduced and an individual is euenergetic i.e. in energy balance at a lower weight(10, 11). Several IER regimens have been developed which attempt to overcome these issues i.e. to promote maintained dietary adherence and weight loss as well as potential additional metabolic benefits beyond those seen with weight loss. Intermittent diets which include periods of limited energy intakes interspersed with normal ad lib eating have gained in popularity in the past decade. The most popular methods involve a 5:2 diet with two consecutive or non-consecutive days of low energy diet (500 – 850 kcal/ day) and 5 days of normal eating each week, or alternate day fasting (ADF) which usually involves 3-4 days of low energy diet (500 – 650 kcal/day) interspersed with days of normal eating across the week. Also, time restricted eating (TRE) where individuals fast for between 12 to 18 hours and eat in a 6–12-hour window each day. TRE aims to restrict energy intake and align eating with the circadian rhythm. TRE usually involves ad lib eating during the eating window (adlib TRE), although some research has tested an energy restricted TRE in which the daily energy restricted diet needs to be eaten in a defined eating window rather than across the day (energy restricted TRE). The most popular IER regimens are summarised in Figure 1.

IER is also popular amongst people who are a healthy weight who are motivated by the purported health benefits of IER for health and disease prevention. A recent US survey of 3000 Americans aged 18 – 80 years including 17% with overweight obesity reported IER as the third most popular diet practiced by 13% of the population(12).

This paper will review evidence for potential benefits and/or harms for each of these three popular IER regimens amongst women who are living with overweight or obesity or who are a healthy weight. Also the potential utility of these diets for managing women’s health conditions will be explored.

## Weight loss and metabolic effects of IER compared to DER in subjects with overweight and obesity

A recent meta-analysis summarised studies which have randomised people with overweight/obesity with or without type-2 diabetes to an IER (either 5:2 or ADF) or an energy matched DER (13). The review included 11 randomised controlled trials (RCTs) and 850 participants, 67% were female and 33% were male. In this review IER achieved slightly better weight loss (7%) compared with DER (5%) in short-term studies (<6 months), but weight loss was comparable between IER and DER in longer-term studies (both ~5–6%)(13). These results suggest superior short-term adherence and weight loss success with IER compared to DER. However, this is not sustained, suggesting IER is hampered by the same behavioural and physiological drivers which promote weight regain with DER i.e. satiety, hunger, reward, adipose immune cells, adaptive thermogenesis and reduced lipolysis and lipid oxidation lipid metabolism(14).

TRE can either ad lib TRE which has an eating window but no prescribed energy restriction, or an energy restricted TRE diet with a prescribed eating window. Ad lib TRE diets typically reduce energy intake by 200-300 kcal/day and are associated with a modest weight loss of 3% in short-term studies(15) and ~ 1% in longer-term studies(16). Studies of TRE diets vs matched energy restricted diets consumed across the day have reported both superior(17) (18) or equivalent(19, 20) weight loss with TRE. These data do not confirm superiority of TRE alongside energy restriction, but show that an eating window can sometimes increase adherence and weight loss with DER.

A further question is whether IER confers additional weight independent effects on metabolic health evidenced by markers including blood pressure, lipids and insulin resistance. IER may have short-term beneficial effects on these markers during the repeated spells of energy restriction each week. In addition, there could be more sustained beneficial effects across the week if for example there were preferential reductions in body fat and ectopic fat stores with IER vs DER. Current evidence does not however support this assertion. Shubel et al reported equivalent weight loss parallelled with proportional reductions in visceral and subcutaneous fat stores with IER vs DER(21). Cioffi et al reviewed 11 RCTs of 5:2, ADF (not TRE) studies including 630 patients (range 8 – 24 weeks) and concluded there were small favourable differences in metabolic markers with IER vs DER (22). These effects were modest and of doubtful clinical significance i.e. IER had 0.05 mmol/L (3%) higher high-density lipoprotein and (15% greater reductions in insulin) compared to DER. These beneficial effects were mainly reported in short-term studies and may simply reflect slightly greater weight loss in these studies. Also, two of these studies involving 223 of the 630 (34%) of participants were testing an intermittent low carbohydrate diet which may have superior glycaemic effects(22).

Studies of TRE have reported modest improvements in some glycaemic parameters which are largely driven by reduced energy intakes and weight loss. Some potential weight independent metabolic benefits have been reported when TRE has an early eating window and avoids the adverse metabolic effects of late-night eating. Whilst promising, early TRE can be difficult to integrate into family / social lives(23).

## Weight loss and dietary adherence with IER in women vs. men

Lower dietary adherence and weight loss across a range of behavioural weight loss interventions have been reported amongst women vs. men, and amongst younger vs older subjects(24) and amongst parents with children living at home(25). Barriers to adherence in younger women include competing demands on time, stress, multiple role expectations, fatigue, limited family support and often unhealthy home food environments which include high energy foods, snacks and drinks(26).

Consistent with this data adherence and weight loss success with 12 weeks of ADF has been reported to be lower amongst premenopausal women (−4.6 ± 3.2%) compared to postmenopausal women (−6.5 ± 3.2%) and men (−6.2 ± 4.4%)(27). Similarly, Barnowsky et al reported lower weight loss with 6 months of ADF amongst premenopausal (–6.0 ± 1.1%) versus postmenopausal women 11.6 ± 2.3%) (28).

In contrast to this, Schroor undertook a systematic review and meta – analyses of 28 RCTs of 5:2, ADF and TRE diets(29). The review concluded the three different IER diets resulted in comparable weight loss and cardiometabolic risk markers change compared with DER diets. However, a sub-group analysis in 7 studies which involved women only (n = 507) showed IER had greater effects on reductions in body weight, body fat and waist circumference than DER. Body weight (Weighted mean differences WMD –1.01 kg; 95% CI: –1.52 to –0.50), body fat (WMD: –1.08 kg; 95% CI: –1.68 to –0.48;), and waist circumference (WMD: –1.40 cm; 95% CI: –2.64 to –0.15). No significant differences between IER and DER were observed in studies with men only or mixed cohorts. Caution is required when interpreting cross study comparisons. The findings in women may reflect different features of the IER and DER regimens and study design in the women only studies, rather than being evidence of a gender specific effect of IER.

Cyclic changes in hormones in premenopausal women are likely to influence appetite and energy expenditure and dietary adherence across each month. Energy intake is often increased in the luteal phase due to cravings for high fat and / or carbohydrate foods (30), making this a potentially challenging time for adherence to a low energy diet for some women. Adherence to the different IER diets across the menstrual cycle is not known. However a menstrual cycle adapted DER weight loss programme which attempted to align with these cyclic variations has had limited success compared to a standardised DER across the month(31). Premenopausal women are reported to have a greater lipolytic response and higher plasma free fatty acids with extended overnight fasting compared to men and postmenopausal women which has a negative impact on postprandial glycaemia, summarised in (32). However the clinical significance of this normal physiological response to fasting is unclear.

## Potential harms of IER in women

### Effects of IER on Fat Free Mass

One concern is whether IER leads to greater loses of fat free mass (FFM) for a given weight loss than seen with DER. In people with overweight / obesity around 25% of weight loss with daily energy restricted diets is loss of FFM(33). IER could lead to greater reductions in FFM for a given weight loss as a result of spontaneous decreases in physical activity during energy restricted periods(34), insufficient protein intakes(35),or sub-optimal regularity of protein intake to optimise muscle protein synthesis(36);the latter being particularly relevant with TRE. Greater losses of FFM with energy restriction are seen amongst subjects with lower fat mass i.e. lean compared to those with overweight/obesity, men compared to women, and alongside more severe energy / protein restriction(37), and in older subjects(38).Weight loss studies with IER amongst women with overweight / obesity have shown that reductions of FFM align with the weight loss achieved with comparable reductions of FFM per kg body weight reduction to DER as summarised in Table 1(17,18,19,69,70,71,72,73,74)

There are no data on the effects of IER on FFM in cohorts of healthy weight women who will be more susceptible to reductions in FFM than women with overweight or obesity. However, several studies report large reductions of FFM in groups of lean men and women exposed to ADF with alternate day 24-hour fasts. Heilbronn et al studied 8 women and 8 men for 22 days who experienced a weight loss of 2.5%, of which 57% was FFM (39). Likewise, Templeman et al compared the effects of this ADF with an energy matched DER over 3 weeks in 12 lean women and 12 lean men. Mean (SD) weight loss and % of weight loss as body fat with DER were -1.91 (0.99) and 92% compared to -1.60 (1.06) and 46% for IER(34). These data highlight a potentially lower FFM retention amongst healthy weight subjects undertaking IER.

There are few data of the effects of IER on FFM amongst postmenopausal women who are at higher risk of developing sarcopenia(40). In the absence of data, it is prudent to advise adequate protein and exercise alongside IER diets. Exercise is well known to attenuate loss of FFM with energy restricted diets(38). This has been reported alongside an ADF diet (mean age 45 years, 81 women, 3 men)(41). Two relatively small studies have examined whether exercise attenuates reductions in FFM with IER. Cooke et al reported that 10 minutes of sprint exercise three times a week was not sufficient to attenuate FFM loss that occurred alongside an intermittent 5:2 diet (mean age 35 years, 8 women, 3 men)(42). However a study young trained women (13 TRE 13 control diet aged 18–30 years), reported that TRE eating in a 7.5 hour window did not compromise accretion of FFM alongside a resistance exercise and high protein diet compared to consumption of regular meals throughout the day(43).

### Effects of IER on bone health

Weight loss with energy restricted diets in individuals with overweight or obesity can reduce bone quantity, bone density and bone quality. The latter is already compromised in subjects with obesity. Bone effects may be partly through reduced mechanical loading at a reduced weight, and may also relate to increased bone marrow adipose tissue and associated cytokine production and adipokines and reduced osteoblast formation(44). Weight loss has been associated with reductions in total hip bone mineral density (BMD), but not lumbar spine BMD(45). BMD reductions of approximately 1-1.5% in weight loss studies are comparable to annual losses in older women, which have been associated with a 10% to 15% increase in fracture risk(45).

The effects of 5:2, ADF and TRE on bone health are not known, nor whether they differ from those of DER. IER could exert detrimental effects on bone health alongside reduced physical activity during the energy restricted spells of intermittent diets. In addition, elevated post-prandial insulin resistance in response to the first post fast meal consumed with IER has the potential to supress concentrations of C-terminal telopeptide (CTX) and osteocalcin. The bone effects of an IER regime will also relate to its nutritional adequacy for bone health (calcium, vitamin D intake)(46).

One of the few data on IER and bone reported that 6 months of ADF or DER both resulted in a weight loss of 8% and that neither diet was associated with reductions in total body dual energy x-ray absorptiometry (DXA) measured bone mineral density, or in circulating bone turnover markers osteocalcin, bone alkaline phosphatase or CTX(28). This study has limitations and is likely to be underpowered for these bone measures, and it did not collect specific hip/spine BMD responses. Powered research of the bone effects of IER are required using validated bone end points i.e. hip / lumbar spine BMD, bone microstructure and fracture risk(46).

### Reproductive hormone levels

Energy restriction may disturb regularity of the menstrual cycle and fertility. Menstrual cycle disturbances i.e. shortened luteal phase, anovulation and / or oligomenorrhea (cycle length 36–90 days, have been observed amongst healthy weight eumenorrheic women aged 18–30 years when exposed to daily energy deficits of between 470-810 kcal day (22-42% energy restriction) over 4 menstrual cycles(47). Energy restriction can suppress the menstrual cycle through inhibition of gonadotropin-releasing hormone (GnRH) pulsatility. There are few data on the effect of IER on reproductive hormones and menstrual cycle function. One study of a 5:2 diet amongst premenopausal women with overweight / obesity has shown average cycle length across the dietary intervention to be slightly longer with IER than with DER, 29.7 (3.8) days vs 27.4 (2.7) days. However there were comparable increases in sex hormone binding globulin (SHBG)+14% vs +6% and reductions in free androgen index -6% vs -10%. The increased cycle length may reflect a slightly longer follicular phase when undertaking IER.However, the clinical significance of this observation is not known. For example research to date has not linked menstrual cycle length with risk of breast cancer(48).

### Intermittent diets and eating disorders

Another frequently cited concern is whether IER could trigger or exacerbate disordered eating. These concerns are based on a theoretical risk and cross-sectional studies which report higher eating disorder scores amongst populations undertaking IER. For example, a recent survey amongst 2762 Canadian adolescents and young adults aged 16–30 years (1477 women, mean age 23, 40% who consider themselves overweight) reported that 47% had engaged in IER in the past 3 and 12 months(49). The majority of these had undertaken TRE (80%), 9% ADF and 11 % other IER regimes. The group reporting IER had higher scores for overeating, loss of control, binge eating, vomiting, laxative use, compulsive eating than those not undertaking IER(49). Likewise, a survey which included 40 women undertaking time restricted eating (fasting for > or = 16 hours / day, mean age 33, mean BMI 27kg / m^2^) reported that those engaging with IER had higher eating disorder scores for binge eating, vomiting and laxative use than community and clinical norms(50). Approximately a third of this cohort reported eating disorder symptom scores above the diagnostic cut offs. These cross-sectional studies do not allow us to ascertain the direction of the relationship between IER and eating disorders. It is likely that IER is not a cause of these symptoms, but that those with eating disorder traits may be more likely to undertake IER. It is also possible that individuals with eating disorder traits may be attracted to volunteer for studies which focus on IER. For future studies it would be prudent to conduct baseline screening using validated eating disorder scales (51) so these subjects can be excluded from studies and directed to appropriate support where relevant.

In contrast, prospective intervention studies of IER in those with overweight and obesity have shown either no change or reductions in eating disorder scores alongside weight loss. A systematic review of 4 studies of TRE (194 participants, 71% women) concluded TRE had neither beneficial or adverse effects on disordered eating(52). Whilst an 8 week ADF intervention amongst women with overweight and obesity reported reduced scores for depression, binge eating and concern about body size/shape assessed with the Multidimensional Assessment of Eating-Disorder Symptoms alongside mean (SD) % weight loss of 4.2 (0.3) %(53). The lack of effect or reductions in eating disorders reported with IER is consistent with reports with weight loss from other behavioural weight management programmes(54).

### IER and sleep

Sleep quality is a key factor for health and well-being. Women may be more predisposed to disturbed sleep than men associated with fluctuating hormone levels. During the luteal phase women can experience more daytime sleepiness, decreased sleep efficiency and difficulty initiating sleep. Sleep disturbance is well documented during the menopause and reported by around 60% of women. This hormonal predisposition can be exacerbated by social factors with many working women undertaking domestic tasks and childcare(55).

Intermittent diets have the potential to have either beneficial or adverse effects on sleep quality. TRE may have a beneficial effect on sleep if the eating window avoids bedtime eating /snacking which can disturb sleep (56). Alternatively, IER regimens with an overall energy restriction may disturb sleep if people experience hunger at bedtime and during the nighttime(57).

A recent review of TRE and sleep reported no effect of overall sleep quality assessed with the Pittsburgh Sleep Quality Index (PSQI), but it included some reports of reduced sleep efficiency with both a late TRE (eating window 12.00 – 8.00 pm) and an early TRE (eating window 7.00 am – 3.00 pm), and reduced sleep duration and sleep onset latency with an early TRE (eating window 7.00 am–3.00 pm)(58). Studies of ADF in all female(59) and predominately female cohorts (81%)(60) have shown no impact on sleep quality assessed with the PSQI. This aligns to data from a range of behavioural diet and exercise weight loss interventions using DER showing no effects on sleep(61). Improved sleep with weight loss in these studies may not be seen since many participants already have good baseline sleep scores (58,59,60). One study reported that TRE can reduce sleep disturbance amongst shift workers with circadian rhythm misalignment and disturbed sleep.(62) Although it did not impact on the other measurements of sleep quality i.e. latency, daytime dysfunction, efficiency, overall quality, need for medication. Future studies should focus on the effects of IER in those with circadian rhythm misalignment and explore whether TRE has potential adverse effects on sleep in those with good baseline sleeping patterns.

### Intermittent diets and Polycystic Ovary Syndrome

An estimated 3.5% of women in the UK have Polycystic Ovary Syndrome (PCOS), which is increasingly linked to increasing rates of overweight and obesity(63). Women with PCOS have hyperandrogenism, sub-fertility and are at increased risk of type-2 diabetes and cardiovascular events. First line treatment involves diet, weight loss and exercise. Li et al tested the effects of 5 weeks of an 8hr TRE amongst 18 premenopausal women with overweight/obesity and PCOS(64). There was a weight loss of 2% over 5 weeks alongside reductions in testosterone (9%) increased SHBG (2%) and decreased free androgen index (26%). These positive results provide some evidence that TRE is a potential weight loss diet for women with PCOS. However, the reported hormonal effects are in line with those expected alongside the weight loss seen and do not verify a weight independent effect of TRE for those with PCOS.

### Intermittent diets and premenstrual syndrome

Weight control is a potential strategy for management of premenstrual syndrome (PMS) (65). Hooshier et al conducted a small short-term trial assessed the impact of ADF (alternate days of 75% energy restriction and consuming estimated energy requirements) compared to a matched DER on premenstrual symptoms amongst 60 women with overweight or obesity(66). The ADF diet group reported reductions in some PMS traits i.e. mood lability and expressed anger, but no global reduction in PMS scores. These beneficial changes were seen alongside greater percentage weight loss in the ADF group vs DER (-6.7% vs -3.7%). The study shows greater short-term weight loss success with ADF vs DER in this population but is unable to conclude there is a weight independent effect of ADF on symptoms of PMS.

### Intermittent diets and gestational diabetes

Gestational diabetes (GDM) occurs in between 1 and 25% of pregnancies worldwide affecting an estimated 16% of pregnancies in the UK(67). Rates are rising linked to higher levels of obesity and maternal age. GDM and poor glycaemic control lead to maternal and neonatal complications i.e. macrosomia, shoulder dystocia, neonatal hypoglycaemia and/or hyperbilirubinemia, preterm delivery, caesarean-sections, preeclampsia, and stillbirth. Diet and exercise are first line therapies for the management of GDM (68). Metformin and / or insulin are utilised if blood glucose targets are not met by changes to diet and exercise. UK NICE guidance promotes healthy eating and promotes low glycaemic index foods and physical activity but has no specific guidance for energy restriction. Other countries advocate carbohydrate and energy restriction, and optimal timing of meals and many include targets for limiting gestational weight gain (GWG). There are no specific targets for GWG for GDM. Many GDM guidelines follow the Institute of Medicine Guidelines (USA) for GWG which recommend lower weight amongst women with overweight or obesity i.e. healthy weight women weight (BMI 18.5 to 24.9 kg/m^2^) should gain 11.5 to 16 kg (25 to 35 pounds) during pregnancy. Overweight women (BMI 25 to 29.9 kg/m^2^) should gain 7 to 11.5 kg (15 to 25 pounds) and obese women (BMI greater than 30 kg/m^2^) should only put on 5 to 9 kg (11 to 20 pounds) (68). A range of daily dietary approaches have been studied for the management of GDM including low-GI diets (limiting refined and promoting complex carbohydrates), low carbohydrate diets or modest energy-restriction (1800 Kcal/day)(68).

However, there is currently no strong evidence to recommend one dietary regimen to improve outcomes in GDM. IER has potential utility for the management of GDM. GDM is a strongly associated with obesity and insulin resistance and is considered a form of evolving Type 2 diabetes. IER has been associated with greater or equivalent reductions in weight and hyperglycaemia in patients with Type 2 diabetes when compared to DER(22, 69), and so could potentially also have benefits for GDM. Dietary management of GDM typically requires a 10–12-week dietary intervention in the third trimester. Since 5:2 or ADF diets appear to have a greater adherence and weight loss than daily diets in the short-term (<3 months)(13), they could have superior adherence to DER in the required short-term treatment period.

Our team are currently undertaking a feasibility study (n = 48) to test the safety, feasibility and acceptability of a 5:2 diet between diagnosis of GDM and delivery in our MIDDAS – GDM study (Trial Registration Number: NCT053440660). The 5:2 diet involves 2 non-consecutive days of 1000kcal which includes 100g low-GI carbohydrate and 70g of protein, and 5 days/week of the NICE healthy eating low-GI diet and physical activity recommended for the best NHS care group(70).

The benefits of a 5:2 diet vs DER has been tested over 12 months amongst non-pregnant women with a history of GDM. The 5:2 diet included 2 non-consecutive days of 500kcal to include 45g low-GI carbohydrate and 50g of protein and 5 days / week of their habitual diet vs. a daily 25% energy restricted diet which typically included 1500 kcal (6000 kJ) per day (170 g carbohydrate, 110 g protein) (71).There was an equivalent high drop-out (49%) and modest weight loss both groups (IER, 5.0 ± 5.4%; DER, 3.5 ± 5.6%; P = 0.3). These results reflect the challenge of weight control within the GDM population and that this challenge was not overcome by those attempting to follow IER.

## Conclusion

Research findings and gaps in research of IER and DER amongst women is summarised in Table 3. IER (5:2 or ADF) diets are an equivalent (but not superior) approach to DER for weight loss Table 2 and managing weight related metabolic conditions for women with overweight and obesity. Adherence to behavioural weight control interventions can be a particular challenge for younger women, which is seen with both DER and IER diets. IER has some theoretical harms for women affecting reproductive health, bone health, disordered eating, and sleep, but there are few data to support or refute these.

Weight loss with ADF and TRE have respectively improved symptoms of polycystic ovarian syndrome and premenstrual syndrome but there is no evidence of weight independent effects of IER on these conditions. Thus, IER is a possible weight loss strategy for these conditions but it is not currently advocated as specific dietary startegy for treating / managing these conditions. IER is a popular diet amongst women who are healthy weight but there are few data to inform its potential benefit or harms which needs to be a focus of ongoing IER research.

## Figures and Tables

**Table 1:** Loss of weight and fat free mass amongst women undertaking intermittent energy restriction versus daily energy restriction

Reference	Cohort Sample size Age BMI	Body composition measurement	Duration/ weeks	IER/ DER regimens	DER		IER	
					Weight loss-Kg	% weight loss as FFM	Weight loss-Kg	% weight loss as FFM
Alternate day fasting								
Hutchison et al 2019 (72)	N = 88 (All female) Age 50 ± 1 years, BMI 32.3 ± 0.5 Kg/m^2^	DEXA	3 week	Both 30% energy restricted diets: ADF 70% ER & energy requirements on non-fasting days DER 30% ER	-3.9 (0.4)*	19.0 (6%)*	-5.4 (0.5)*	19.0 (7.0) *
Beaulieu et al 2020 (73)	N = 54 (All female) Age 35 ± 11 years, BMI 29 ± 2.5 Kg/m^2^	Air displacement plethysmography (Bod Pod)	12 week	ADF alternate days 75% ER/ adlib DER 25% ER	-4.0	32.5%	-5.3	24.5%
Coutinho et al 2017 (74)	N = 35 (79% female) Age 39.4 ± 11.0 years BMI 35.6 ± 3.2 Kg/m^2^	Air displacement plethysmography (Bod Pod)	12 week	Both 33% energy restricted diets ADF alternate days 550 – 660 kcal formula diet & prescribed food based diet DER 33% ER	-8.2	23.2%	-13.9	20.1%
Bowen et al 2018 (75)	N -164 (81% female) Age 40.0 ± 8.3 years BMI 35.7 ± 5.9 Kg/m^2^	DEXA	16 week	ADF alternate days 1200 kcal and 600 kcal meal replacement and food diet & 1 ad lib eating day DER 1200 kcal meal replacement diet and prescribed food diet	-12.4	15.3%	-12	14.0%
5:2 diet								
Harvie et al 2011(76)	N = 107 All premenopausal women Age 40.1 ±4.1 years BMI 30.7(5)Kg/m^2^	Single frequency bioelectrical Impedance (leg to leg)	26 weeks	5:2 :75% ER 2 days (650 kcal) DER 25% ER	-6.4 (-7.9 to -4.8)^**^	21.0%	-5.6 (-6.9 to - 4.4)^**^	21.0%
Harvie 2013 et al (77)	N = 115(All female) Age 45.6 ± 8.3 years BMI 29.7 ± 4.1 Kg/m^2^	Multi-frequency bioelectrical impedance 5 kHz / 50 kHz / 250 kHz / 500 kHz	16 weeks	5:2 75% ER (650 kcal < 50 g carbohydrate) DER : 25% ER	-5.5	36·0 (26·4- 41·3) %^**^	-3.8	29·3 (25.0-38·1)* %*
Time restricted eating								
Lin 2022 et al (17)	N = 63 (All female) Age 50.1 ± 7.5 years BMI 25.9 ± 3.7 Kg/m^2^	Multi-frequency bioelectrical impedance 1, 5, 50, 250, 550, 1000kHz	8 weeks	TRE: 1400 kcal (8 hour eating window) DER: 1400 (no time restriction) Both groups 8 × 30 minute exercise sessions	-1.6	12.5%	-2.7	26.0%
Jamshed et al 2022 (18)	N = 90 (80% female) Age 43.0 ± 10 years BMI 40.1 ± 6.6 Kg/m^2^	DEXA	14 weeks	TRE (500 kcal/d below their resting energy expenditure) +8 hour eating window DER – 500 kcal deficit diet no time restriction Both groups advised to exercise 75 -150 mins/ week	-4.0	26.0%	-6.3	22.0%
Thomas et al 2022(19)	N = 81 (85% female) Age 38.0 ± 8.0 years BMI 34.1 ± 5.7 Kg/m^2^	DEXA	39 weeks	TRE (33% ER + 10 hour eating window within 3 hrs of waking DER : 33% ER no time restriction Both groups advised to exercise 150 mins/ week	-5.1	21.6%	-6.2	24.2%
Ribiero et al 2022 (20)	N = 24 (83% female) Age 33.0 ± 8.7 years BMI 31.7 ± 8.6 Kg/m^2^	Multi-frequency bioelectrical impedance 20, 100 kHz	8 weeks	TRE (20% ER+8 hr eating window) DER (20% ER no time restriction) Both groups active: 3x 20 min/ week aerobics exercises + resistance training + exercises to balance and proprioception.	-6.3	22.2%	-5.7	0.0%

FFM = fat free mass DEXA =Dual energy X -Ray absorptiometry ADF = alternate day fasting DER daily energy restriction IER intermittent energy restriction TRE = time restricted eating

* mean(SD)

** mean (95% confidence interval)

**Table 2:** Summary of research findings and gaps in IER research for weight control and metabolic health for women

	Effects in women who are overweight and obese		Effects in women who are a healthy weight	
Outcome	Current evidence	Research gaps	Current evidence	Research gaps
Weight loss	ADF/ 5:2 diets are superior to DER in short-term studies(< 6 months) but equivalent inlonger-term studies (≥ 6 months) Dietary adherence & weight loss success is reduced with ADF/ 5:2 diets and DER amongst younger women.	Need effective strategies for weight management with DER or IER for young women	No evidence	Are there potential benefits of IER for metabolic health or weight gain prevention?
Potential harms of IER				
Bone health	Limited data	Require powered research with validated bone end points i.e. hip / lumbar spine BMD, bone microstructure and fracture risk	No evidence	What are the effects of IER on bone health in women who are a healthy weight?
Reproductive hormones	No evidence of harm but minimal data	Studies of IER should collect this information	No evidence	Does IER affect reproductive hormones in lean individuals who are more susceptible to the hormonal effects of energy restriction?
Eating disorders	Either no effect or beneficial effect amongst overweight/ obese alongside weight loss	Studies of IER should collect this information	No evidence	Does IER lead to disordered eating in healthy weight individuals?
Sleep quality	Some reports of decreased sleep efficiency and sleep onset latency with TRE. No effect on sleep with ADF which aligns with findings from DER weight loss interventions.	Need more evidence in those with circadian misalignment and those with current good sleep. Also, populations at high risk of disturbed sleep i.e. menopausal women	No evidence	What are the effects of IER on sleep in women who are a healthy weight?
Benefits to specific women’s health conditions	Weight loss with TRE improved PMS Weight loss with ADF improved PCOS These IER diets are feasible alternative weight loss diets in these populations. However, there is no evidence of weight independent effects of IER on these conditions The effects of IER on GDM is under study.	Need randomised trials to compare the effects of IER to DER for these conditions	No evidence	Require more research of the effects of IER on specific women’s health conditions amongst women who are a healthy weight

PMS = Premenstrual syndrome PCOS =polycystic ovarian syndrome GDM =gestational diabetes IER= intermittent energy restriction DER= Daily energy restriction TRE= Time restricted eating ADF = Alternate day fasting BMD = Bone mineral density
